# Improved standardization of transcribed digital specimen data

**DOI:** 10.1093/database/baz129

**Published:** 2019-12-09

**Authors:** Quentin Groom, Mathias Dillen, Helen Hardy, Sarah Phillips, Luc Willemse, Zhengzhe Wu

**Affiliations:** 1 Department of Collections, Meise Botanic Garden, Nieuwelaan 38, 1860 Meise, Belgium; 2 Department of Life Sciences, Natural History Museum, Cromwell Road London SW7 5BD London, UK; 3 Department of Collections, Royal Botanic Gardens Kew, Richmond TW9 3AB London, UK; 4 Department of Entomological Collections, Naturalis Biodiversity Center, Darwinweg 2, 2333 CR Leiden, The Netherlands; 5 Finnish Museum of Natural History, University of Helsinki, Unioninkatu 44, 00170 Helsinki, Finland

## Abstract

There are more than 1.2 billion biological specimens in the world’s museums and herbaria. These objects are particularly important forms of biological sample and observation. They underpin biological taxonomy but the data they contain have many other uses in the biological and environmental sciences. Nevertheless, from their conception they are almost entirely documented on paper, either as labels attached to the specimens or in catalogues linked with catalogue numbers. In order to make the best use of these data and to improve the findability of these specimens, these data must be transcribed digitally and made to conform to standards, so that these data are also interoperable and reusable.

Through various digitization projects, the authors have experimented with transcription by volunteers, expert technicians, scientists, commercial transcription services and automated systems. We have also been consumers of specimen data for taxonomical, biogeographical and ecological research. In this paper, we draw from our experiences to make specific recommendations to improve transcription data. The paper is split into two sections. We first address issues related to database implementation with relevance to data transcription, namely versioning, annotation, unknown and incomplete data and issues related to language. We then focus on particular data types that are relevant to biological collection specimens, namely nomenclature, dates, geography, collector numbers and uniquely identifying people. We make recommendations to standards organizations, software developers, data scientists and transcribers to improve these data with the specific aim of improving interoperability between collection datasets.

## Introduction

There are an estimated 1.2–2.0 billion specimens in the world’s herbaria and museums (1). Biological collection specimens are distinct categories of biodiversity occurrence record. They are scientifically important because, unlike field observations, specimens provide direct evidence for the occurrence that can be further studied and validated. The core data of a biological occurrence record are the identity of the taxon or taxa ‘what’, the date of the occurrence ‘when’ and the location ‘where’. Researchers use specimens for many kinds of studies and for many such applications, these data are enough (2, 3). If they are not, there are many additional pieces of information that are associated with specimens, including other data related to the collection event, the collection location, the specimen’s nature or preparation and the subsequent history and use of the specimen.

Collection specimens share some characteristics with those observations that are supported by photographic evidence, as they both provide evidence for the observation and the possibility of extracting additional information. However, the potential preservation of DNA and the accessibility of morphological and microscopic characters make specimens particularly valuable from a scientific perspective. In recent years, photographically supported observations are being created in their millions, notably by online platforms such as iNaturalist (www.inaturalist.org/), Observation International (observation.org) and Biodiversidad Virtual (www.biodiversidadvirtual.org). However, an important distinction with specimens is that these platforms capture data associated with an observation digitally, directly at the point of creation, whereas the vast majority of specimens in collections are first documented on paper. Digitizing these data from paper, in a useable format, is an ongoing challenge for collection holding facilities, such as museums, universities, botanic gardens, field stations, etc. Harmonizing these digital data to make them semantically interoperable is an even bigger challenge. Semantic interoperability means that data fields from different datasets have a common meaning and share compatible definitions, even if they might have different property names.

Specimens are used to support many biological studies, but perhaps their foremost use is in taxonomy and nomenclature, where they are used as type specimens to link a scientific name to a taxon. The details of such type material are published in taxonomic literature, where they are generally referenced by the institution they belong to, their collector and sometimes their collector number. As a result of this use, the identity of the person or persons who collected the specimen ‘who’ and the collector number ‘which’ is of similar importance to the what, when and where of the collection event.

Often, a collector will document additional information pertaining to the collecting event in field notebooks, scientific publications and, nowadays, even in mobile telephone apps. The ‘who’ and ‘which’ are often critical to link this additional information back to the physical specimen. At least in part as a result of this additional information, specimens have also found use in subjects as diverse as evolution, genetics, climate change impacts, history of science, morphology, medicine, social justice and ecology (4–10).

The data standards ABCD and Darwin Core were created to organize and catalogue data on biological collection specimens (11, 12). The use of these standards has since been expanded to accommodate data on other types of biological observation, including those associated with photographs (13), ecological surveys (14), species checklists (15) and geological specimens (16). Darwin Core, in particular, has received widespread use through its adoption by the Global Biodiversity Information Facility (17).

These standards have been widely adopted not only because the community recognizes the need for data interoperability but also because these data standards are flexible. Some recommendations exist in the standards as to the content of fields, but few terms have controlled vocabularies that are actively enforced or well documented. This makes these standards comparatively easy to conform to, allowing for a wide degree of interpretation on how a particular field might be used (18). This flexibility is an advantage from the perspective of data publishers who both want to conform to a standard and must consider the local needs, priorities and resources. On the other hand, users of data, particularly of aggregated resources, find the malleability of these standards a significant hurdle to use of these data. A time-consuming data ‘cleaning’ exercise must be conducted before any data can be used (19, 20). This often entails a large amount of manual work and, although this work results in standardized data, there is rarely a mechanism to return these corrected and standardized data to their source. Seldom has even partially automated data cleaning been achieved (21). Therefore, the dream of data interoperability has, at best, only been partially achieved, and more work is required on standards and processes to improve upon the *status quo*.

The broad scope of biodiversity occurrence data makes complete semantic interoperability of data fields extremely difficult. Indeed, for some types of data, it is perhaps undesirable to enforce too strict a standard. The standard would either have to be over-complicated or overly restrictive. However, specimen data often represent a well-defined subset of occurrence data, with a fairly narrow scope of potential elements and uses. For this reason, there is merit in refining the use of occurrence standards specifically for specimens.

There is a demand for some conformity not only in the fields used but also for controlled vocabularies that restrict the allowable values for these fields. In the case of herbarium specimens, the Apple Core project has made recommendations for how Darwin Core fields should be used (applecore.biowikifarm.net/wiki/Main_Page). More generally, there are other publications that make specific recommendations for biodiversity occurrence data, including for specimens (22–25).

Here, we summarize several studies we have conducted on the digital transcription of biological specimen data from their associated specimen labels. We draw on trials we have conducted on the automated and manual transcription of specimen labels (26, 27). We have also investigated how specimen data are shared by institutions and how they store these data in their collection management systems (18). The goal of this paper is to make recommendations to biodiversity data scientists and standards organizations [e.g. Biodiversity Information Standards (TDWG), International Association for Plant Taxonomy, International Commission on Zoological Nomenclature] on how to make biological specimen data more interoperable and easier to use. Most of our assessments are based on experience with mounted herbarium specimens, as the digitization of these kinds of specimens is at a more advanced stage than any other (28). However, most principles concerning data capture and data interoperability are similar across different types of biodiversity and geodiversity collection. The data on woody, zoological, mycological and geological specimens are also captured on labels attached to, or stored in proximity to, the physical specimen. Problems related to external sources such as notebooks and scientific articles are also similar. The what, when, where and who are similarly the core data for these specimens. There are doubtlessly some differences for data interoperability due to the nomenclatural codes and geological classifications, yet the principles are common. We also encourage readers interested in this subject to consult other recommendations on biodiversity data management, such as (22) and (29). Furthermore, the general principles of data management are also apposite (30, 31).

## Transcription considerations

### Verbatim transcriptions, versioning and annotations

#### Verbatim data

Verbatim data constitute the literal texts as they are written on the specimen labels, as opposed to interpreted data that are regularly available in databases. Interpreted data are generally better for the findability of specimens, aggregating data, linking related data and for scientific research (32). However, verbatim data are useful to understand the extent to which the standardized data have been interpreted and can support data cleaning. For instance, an unrealistic collection date can be relatively easily corrected if a correct verbatim transcription of the date is also present. Characteristic syntax, vocabulary and abbreviations in verbatim data can be a clue to the identity of the specimen’s collector and the time of their life when they made the collection. Verbatim data can be searched, and they are useful when a transcriber cannot interpret part of the label text, leaving a verbatim transcription for someone else to build upon later.

**Figure 1:**
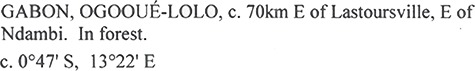
An example of location information on a herbarium sheet label. This text can be entered into a database in several ways, even if the goal is to transcribe it in a verbatim manner. For example, should the dwc:verbatimLocality contain the country ‘Gabon’, the province ‘Ogooué- Lolo’ and the habitat ‘In forest’? Transcribers may decide to distribute parts of this text into the fields dwc:country, dwc:stateProvince, dwc:locality, dwc:verbatimLocality and dwc:habitat or they might choose to transcribe everything literally into dwc:verbatimLocality. Source: http://www.botanicalcollections.be/specimen/BR0000013860288.

Increasingly, an image of the specimen label can be easily consulted or requested and, as a consequence, the importance of verbatim transcriptions is diminishing. However, verbatim transcriptions may also have a new future to train machine learning algorithms that are used to automatically interpret specimen labels. Precise verbatim data are needed as ground truth data to train these algorithms in a way that interpreted text cannot be used, e.g. (33). However, one has to consider that these so-called verbatim data are still, to some extent, an interpretation of the label. They can contain mistakes, but most importantly they also constitute an interpretation by the transcriber of what field a certain verbatim value should fit. For example in Figure 1, some transcribers would list all this text (excluding the coordinates) as a verbatim value for locality, whereas others may distinguish verbatim names for country, county and/or habitat and subsequently split these into different verbatim fields. Spatial arrangement of verbatim text is also important for artificial intelligence strategies, such as machine learning and convolutional neural networks, that hold the promise of finding relationships between and across digital objects documents, specimen labels, field notebooks, etc.

The standards of Darwin Core and ABCD have verbatim terms for some data fields. In principle, there could be a verbatim alternative for any field that might be printed on a specimen label. Yet, such a proliferation of fields could be counterproductive to interoperability of data, particularly if verbatim fields were completed in preference to interpreted fields. Another problem is that these verbatim fields are also used for unstandardized data, such as dates in different formats or country names in different languages. Such data may be verbatim as they are present on the specimen, but they can also be interpretations made using obsolete or bespoke standards, such as a locally devised list of abbreviations for country names, or using external information that is not present on the actual specimen.

Table 1 summarizes use cases for verbatim transcriptions. Not all of these use cases are intercompatible; for example, the use of training data for text recognition (5) requires exact unparsed transcriptions, which is incompatible with the storage of unstandardized yet interpreted data (6).

**Table 1:** A list of use cases for verbatim data, with examples and notes on applications

	Use case	Examples	Application notes
1	Facilitating data cleaning and indicating the degree of interpretation in the standardized fields	Dates that are found to be unlikely or impossible can be easily checked for typos or erroneous transcription	If a digital image of the label is available, then there is less need to check a verbatim transcription for validation.
2	Discovering information hidden in the typography of how text is presented on the label	The syntax of person names can be a clue to the writer’s identity and for linking related specimens	This is unnecessary for most specimens but is valuable for enriching poorly documented specimens.
3	Increasing the findability of specimens.	Where a word, such as a place name, can be read but not understood, then the text can still be found	Original text can be searched in the original language.
4	Accommodating partial or uncertain transcriptions, which would otherwise clutter standardized, interpreted fields	The use of square brackets ([]) and ellipses to indicate uncertainty or a failure to read part of the text	Other transcribers can build on the initial attempt, and it will be clear that the information is present on the label.
5	Providing training and validation source data for automated text capture methods	Automated reading of 19th century handwriting and recognition of symbols used on labels	Finding gold standard training data for algorithms is a common problem.
6	Accommodating data that are not sufficiently standardized for the interpreted field or that fail to comply with the restrictions of the interpreted field	Dates that lack a year or data awaiting interpretation	It is common to find verbatim fields containing data in non-standard formats, yet they are not transcribed data either.
7	Accommodating data following obsolete or bespoke standards	Grid system location codes	When a database is migrated from one system to another, then verbatim fields are used to store old formats.
8	Preserving the original language when interpretation has included translation	Habitats can have some very specific meanings in different languages and they are difficult to translate because there may not be a direct equivalent.	This also improves the findability of specimens written in a different language.

#### Versions as an alternative

An alternative to using verbatim data fields is the use of record versioning. One can imagine many different versions of a specimen record derived from different sources and methodologies. The different sources might be directly from the physical specimen, from literature about the specimen, from a different transcriber, from a field notebook or from duplicate specimens (34). The different methodologies would include verbatim transcription, interpretive transcription, optical character recognition (OCR) or some other form of artificial intelligence. However versions are created, they should be associated with metadata to make their origin clear. It must also be clear to the user which version of a record is suited to their requirements. This implies a standardization of version metadata.

A common approach is to always have the best or recommended version as the default. A similar approach is used in Wikimedia projects and research data repositories, such as Zenodo (zenodo.org), where the latest version is generally the most accurate, up-to-date and complete. However, this may not be the case for specimen record versions, as newer ‘versions’ might be the product of OCR or external sources such as duplicate specimens. These newer versions may be complementary or superior in content to the previous version, but they may also be worse or different in scope (e.g. verbatim transcription for algorithm training vs standardized data for biological research).

#### Annotation as an alternative

Written annotations have a long tradition with specimens, particularly on herbarium sheets, where there is ample space. These annotations can be written on separate labels, on the original label, pinned to specimens or stamped on the mounting sheet. These annotations record identifications, typifications, ownership and other history of the specimen. Specimen annotation also has a digital equivalent (35, 36). Digital annotations could potentially take many other forms, varying from comments concerning individual data fields (e.g. this scientific name is incorrect) to those concerning the whole record or a portion of an image of a specimen. Much like different versions, structured metadata are necessary to understand the context of an annotation, such as when it occurred, who made it and which field or fields it refers to.

There is a considerable overlap between the data that could be maintained as a digital annotation, a version or a verbatim transcription. There is also a danger that digital annotations, versions and verbatim fields could be used inappropriately to add any data to a specimen in an unstructured manner, where structured alternatives exist.

#### Recommendations for standards and software development

Versions, annotations and verbatim transcriptions all overlap in function to express different information about a specimen and its transcribed label data. They are needed because data do change, for example, when a new determination is made or new transcription methodologies are employed. Digital annotations should not be used as a dustbin for any kind of information that cannot be easily updated on a record, due to lack of support for those data in standards and lack of support for versioning in software. We feel that time-stamped and signed versions of digital specimen records are what we should aim for, and annotations should only be used for notes that are intended to be temporary or contain exceptional information. The origins of these data should also be made explicit in metadata so that the methods used to derive the data are clear. Whatever system is used, it has to be simple so that the end user can easily trace the provenance of the information.

#### Recommendations for transcription

Verbatim transcription should be exactly that a literal digital rendition of the text as it is present on the physical specimen. The only exception is the use of square brackets to indicate omissions and uncertainty as described in the following section. However, verbatim sentences containing different types of data must be parsed into core data fields to make them more easily interpretable. When requesting verbatim transcription, consider their downstreams uses.

### Unknown and incomplete data

Data concerning a specimen may not be available for different reasons. The data may have never been recorded on the labels or in registers and notebooks and hence may not be immediately, or ever, knowable. For reasons of speed and cost, label data may have only been partially transcribed, in which case some data might only be available if one has access to either the specimen or an image of the specimen. When prioritizing transcription work, it would be useful to know beforehand what data are available on the labels to be transcribed but often it is not known what is there, only that it might be. Still, there is a critical difference between data that are known to be unavailable and data that might be. Collection managers and funders want to track the progress and costs of digitization, so they need to know which data are yet to be transcribed. Furthermore, research services, such as digitization on demand, can only be offered efficiently if the degree of digitization is known (37).

In addition to missing data, sometimes data are actively withheld by a data provider. Withheld data are currently identified in Darwin Core and ABCD using an information withheld field (dwc:informationWithheld, abcd: InformationWithheld). A downside of this approach is that it is not apparent which information has been withheld from a data field unless the information withheld field is simultaneously consulted. These standard terms in ABCD and Darwin Core have no suggested controlled vocabulary or standard format. So, there is no way to sort through them or evaluate the field automatically.

These esoteric distinctions between different sorts of missing data do not impact many users. If data are missing, it is disappointing for a scientist, but the reasons are moot. Still, some best practices are needed, particularly for monitoring the state of digitization. In a numeric field such as dwc:sampleSizeValue, it is a poor practice to use text, zeros, negative numbers or large numbers as indicators of an empty field or withheld information (24). In these cases, machine-readable metadata are essential to provide information on what blank entries mean and what data are withheld. In text fields, distinct values should be used for data that are known to be unavailable. For example, if no collection date is available for a specimen, a standard value indicating this can be written in the dwc:verbatimEventDate field. A traditional method of indicating this has been the use of ‘S.D.’, an abbreviation for the Latin *sine dato*. Various versions of this can be found, including ‘sd’ and ‘s.d.’ Another complication is partial dates where the year or century is unknown. Such cases are not covered by the ISO standard for dates (ISO 8601), but standardized information on the time of year may yet be relevant.

#### Recommendations for standards

Machine-readable metadata should be available for a standard. This should define the data types and permitted values for each field. All text fields should allow the values listed in Table 2. For certain fields, such as collector, collector’s number and location, allowable synonyms for ‘unknown:missing’ could be ‘s.c.’, ‘s.n.’ and ‘s.l.’, respectively, although these are only loosely standardized (e.g. the known:withheld value can be used for data that have been omitted from publication, for instance, due to policy or conservation reasons).

**Table 2:** A list of terms for missing data values that could be applied to fields in Darwin Core

Missing data terms	Definition	Example
unknown	The information is not digitally available.	Empty value in a digital record of unknown provenance
unknown:undigitized	The information is not digitally available. No attempt has been made to digitize it.	Empty value in a skeletal record to which data still need to be added from the label
unknown:missing	The information is not digitally available. It appeared to be absent during digitization.	A value of S.D. used by transcription platforms to indicate the absence of a date value
unknown:indecipherable	The information is not digitally available. It appeared to be present during digitization, but failed to be captured.	An indication made by a transcriber that they failed to transcribe the information
known:withheld	The information is digitally available, but it has been withheld by the provider.	A georeferenced record for which coordinate data are available but withheld for conservation considerations

The generic unknown indicates that the information is indeed not available.

The additives undigitized, missing and indecipherable allow elaboration as to why the data are unavailable, if this reason is known.

known:withheld indicates that the data are digitally available in a more primary source and could potentially be retrieved after contacting the data provider.

ABCD is currently defined by an XML schema; although there are schemas for Darwin Core, they are not normative and both standards could be more explicit about the allowable values for their fields. Such vocabularies can be implemented following the specifications of TDWG (38).

#### Recommendations for transcription

It is preferable to create a distinction between incomplete and uncertain transcriptions. Square brackets and ellipsis are widespread and commonly used format to add explanations to text and indicate omissions of text and we recommend their use in verbatim transcription (39). The characters ‘[…]’ can be used to indicate incompleteness, whereas any other characters between square brackets identify uncertainty of the transcriber. Do not use question marks or other characters outside square brackets unless they are actually present on the label. These proofreading symbols should never be used with interpreted fields.

#### Recommendations for software developers

The general values of unknown:missing and unknown:indecipherable in Table 2 should be easy to indicate; for instance, using checkboxes or dropdown menus. unknown:undigitized can be used as a default for data properties that were not part of a transcription process, indicating that no attempt was made to capture these data.

### Languages and scripts

Labels, particularly in large international collections, are written in different languages and scripts with accented and non-Latin characters such as the German ß and the Scandinavian Å. A sample of 1800 specimens from nine European herbaria revealed labels written in at least 12 languages (40). These languages may have characters not found on local keyboards and OCR might not be configured to recognize them. Certain symbols, particularly those peculiar to biology, may not be easily recognized or transcribed correctly, such as the characters ±, ♂, ♀, 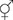, ×, 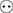 and ⊖.

This problem will be exacerbated for languages that do not make use of the Latin script and for handwritten labels. However, such labels are unlikely to be digitized effectively, or at all, by people unfamiliar with the script (and language). The impact of the language that transcribers are familiar with on their transcriptive behaviour is not clear. Transcribers familiar with the language may be expected to provide more correct transcriptions, as they will be more capable of recognizing the vocabulary and certain grammatical patterns. Transcribers who do not understand the label language will be more likely to transcribe on a verbatim basis, possibly misinterpreting certain terms into the wrong fields or transcribing non-existent words. They may also be more likely to use indicators of uncertainty or incompletion (Section 2.2).

#### Recommendations for software development

Use an implementation of Unicode encoding, such as UTF-8, to facilitate the introduction of non-Latin characters to a verbatim field. For characters that are relatively common on labels but not on keyboards, introduce a tool or widget that facilitates their insertion into the different transcription fields. Where the choice of field entries can be restricted, a dropdown menu is an option for adding entries with non-keyboard characters. Another useful approach could be to allow transcribers to signal the label language. This would serve as an indication of uncertainty due to unfamiliarity with that language and as an invitation for a native speaker to validate the transcription. Such an approach may also make use of image crops with certain symbols in them. These symbols could then be annotated by experts and/or used as training sets for algorithms.

#### Recommendations for transcription

If a verbatim data field is going to be used, then the actual language and script of the text should be used. While it is appreciated, transcribers may be unfamiliar with some scripts—a translation or transliteration is an interpretation of a field.

## Recommendations for core data

As described in the introduction, many different sorts of data can be associated with a biological specimen but the core data are what, when, where, which and who. In this section, we will discuss these core data types in more detail.

### What: taxonomic names

Specimen labels may have mistakes in their scientific names and other legitimate spelling variants, such as abbreviations. Also, many taxon names mentioned on labels are no longer the currently accepted name. Nevertheless, these names must be documented to understand the identity and determination history of the specimen.

Although there is no central name registry for all published taxonomic names, there are some excellent and extensive registries for names. For example, there are the International Plant Names Index (IPNI), MycoBank, Index Fungorum, Zoobank, Index Nominum Algarum, AlgaeBase, Index Nominum Genericorum, The International Fossil Plants Names Index and the Plant Fossil Names Registry. These name registries should not be confused with taxonomic checklists that aim to determine accepted taxa and their synonymy. These registries are lists of published names, together with their authors and publication history.

Neither Darwin Core nor ABCD have a verbatim field for taxon names, so there is no way to record the words on the specimen label unless different versions of the record can be created. Without the option of a verbatim field, therej is little point recording the verbatim data as this will reduce the utility and findability of the data. The scientific name on the label should be interpreted so it can be linked to one of the registries of taxonomic names. This is not recognition that this name is the accepted name of the taxon but a quality control on the existence of the name on the label.

For author names in taxon names, International Commission on Zoological Nomenclature (ICZN, Art. 51) and International Code of Nomenclature for algae, fungi, and plants (ICN, Art. 46) do make recommendation on formats but give such leeway that there is, in effect, little consistency. The ICN (Rec. 46A) does note the use of standard abbreviations of authors that are based upon and maintained by the IPNI (http://www.ipni.org) and Index Fungorum (http://www.indexfungorum.org) (41). However, use of these abbreviations is only a recommendation and no such system exists for zoological names.

Diacritical signs and ligatures are not permitted in scientific names by the ICN (Art. 60.7) and ICZN (Art. 27) so if these do occur on labels, they must be removed. However, this only concerns the Latin portion of the name, not the authorship. In the ICN, it is recommended that the author name is romanized without diacritical signs, but this is only a recommendation and the author’s preference is followed (Rec. 46B).

#### Recommendations for transcription

All scientific names on a specimen should be linked to the stable identifier of that name in a nomenclatural repository. In Darwin Core, this can be done by linking the name to the stable identifier for that name in dwc:scientificNameID. All published names not found in the taxonomically relevant repository should be reported to a repository, preferably with details of its publication.

In rare cases, label names will not be found in names repositories. All names repositories welcome submission of new names and much of the source material is available online in places such as the Biodiversity Heritage Library. Though this might seem like additional work, it can turn a largely useless specimen into the source of scientific information, and it may even be an unrecognized nomenclatural type specimen. In the rare cases where a published name cannot be found in a repository, the name can be linked to its publication in Darwin Core using dwc:namePublishedIn and dwc:namePublishedInID. Occasionally, there are also names on specimens that have never been published. It is better that such names are restricted to a notes field, rather than these names gaining authority by being distributed more widely through data aggregators.

#### Recommendations for software development

In transcription systems, an auto-suggest functionality for taxonomic names based on names registries, such as the ones listed above, is the quickest route to consistency. This can be complementary to a verbatim field.

#### Recommendations to taxonomists, nomenclaturists and nomenclatural repositories

Where not already available, seek to completely document all published scientific names, support the registration of new names and provide stable persistent identifiers for all names. Use standard abbreviations for name authors where they are available and begin the process of standardizing author names for zoology.

### What: nomenclatural types

Nomenclatural type specimens are fundamentally important to biological nomenclature and taxonomy. Type specimens link a published name to a taxon concept. Yet there is no requirement from the international codes of nomenclature to register types, and there is no central repository for typification information. Moreover, there are few examples of collections that know all the types they hold. Even in cases where types are identified and curated in a collection, it is even rarer that the category of type (e.g. isotype, lectotype …) is known. These data and this information deficit are serious impediments to the smooth and efficient working of the codes of biological nomenclature. Names can be accidentally typified multiple times, specimens can be considered lost and description of new taxa can be delayed or made in error.

Nomenclatural types are inseparable from the literature that declared them to be a type. In documenting types, it is essential to include the bibliographical reference of typification. The international codes for nomenclature make recommendations for publications to ensure that type material can be found from the starting point of literature, such as articles 9C and 40A of the ICN (42). However, it is considerably harder from the starting point of a specimen to discover what sort of type it is, where it was typified and whether it is even a type. Only a few years ago, this was largely irrelevant because the main route to specimen data was through the literature. However, now that digitization is making specimen data more widely accessible than typification literature, taxonomists are more likely to find type specimens first and ask the question, as to where it was typified.

#### Recommendations to curators and taxonomists

If a specimen or illustration becomes a nomenclatural type, it should be clearly labelled as such upon publication of the typification and the collection catalogue should be updated with the publication details, particularly the identifiers for the publication, such as a digital object identifier, or an ISSN. If primary- and secondary-type materials are not in the institution where the taxonomist works, they have a responsibility to inform the responsible collection holding institute. This is also a recommendation of the ICZN in articles 72D and 72F (International Commission on Zoological Nomenclature, 1999). However, this is not yet a recommendation of the ICN (42).

#### Recommendations to standards organizations, developers and data scientists

Darwin Core has a Types and Specimen extension and ABCD has a ‘NomenclaturalTypeDesignations’ container element for typification information. Nevertheless, typification data are poorly supported in specimen catalogues and standards lack clear vocabularies for typification data. These data need improvement. Of course, these data also need to be shared openly because types are dispersed across collections, particularly isotypes, paratypes and syntypes.

#### Recommendations to data aggregators

Creators of scientific name aggregations should put more emphasis on typification information, making the links between names, publications and specimens. In aggregated data, the rules of nomenclature and particularly typification can be tested and errors can be identified. In this way, errors in data or typification can be corrected. For example, for any scientific name there should be only one holotype, lectotype or neotype; syntypes should not exist if there is a neotype, isotypes should share the same collecting details and allotypes should not be the same sex as the holotype (42, 43).

### When: dates

**Figure 2:**
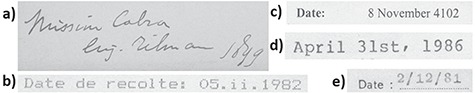
Examples of potential problems encountered while transcribing dates from specimen labels. (**a**) Handwriting difficult to interpret (1849 or 1899). (**b**) Symbolism used can be interpreted differently (5 February or 5 November). (**c**) Impossible but partially true date (correct year was 2002). (**d**) Impossible but likely mostly true date. (**e**) Uncertainty of order of day and month and missing century digits (2 December or 12 February, of 1981 or 1881). All examples from specimens in the Meise Botanic Garden herbarium.

On the face of it, dates are one of the simplest data types to be documented and validated. They are also one of the most important data elements for use in conservation assessments, climate change research and historical studies. Nevertheless, for many reasons they are often the source of confusion and errors [(Figure 2) and see supplementary data 7 of (20)]. Dates on labels can come in a wide range of formats and a wide range of errors or ambiguities can be present. The century is frequently omitted from a handwritten date and numbers can be particularly difficult to distinguish in handwriting, as there are no contextual clues that aid word recognition. Dates may also simply be non-existent (Figure 2d) or ambiguous (Figure 2e).

**Figure 3:**
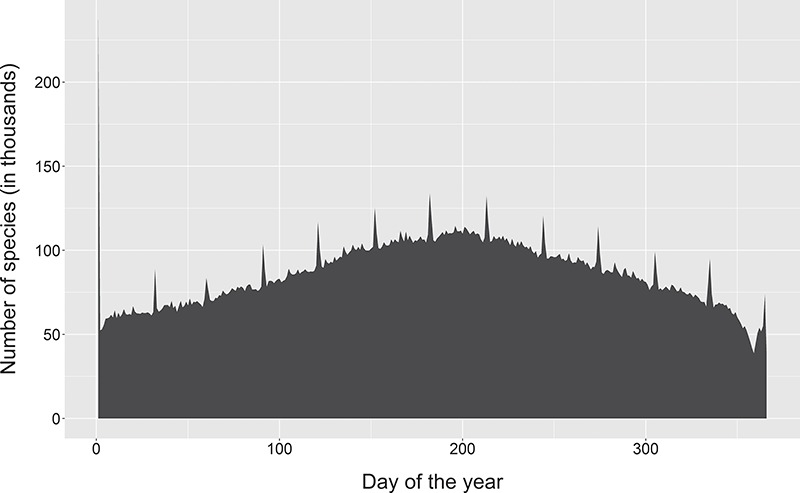
Change in dates of observation for occurrence records on GBIF. Note the 12 spikes corresponding to the first day of each month, with a disproportionately large spike for the first of January. This is more likely caused by many systems, including GBIF itself, storing partial dates as the first day of the month and only using the start date of a date range. Created from a snapshot of GBIF taken on 06 April 2019.

Today, the ISO Standard 1806 is recommended for dates (22). This format can accommodate not only single dates and ranges but also times and imprecise dates. However, there are still many local date formats that have changed with time (Figure 2). The popular spreadsheet program Microsoft Excel has several date-related issues in its basic programming, which can be the cause of corruption of date data. It fails to parse dates before 1900 in its own date format, as it stores dates internally as days since 1 January 1900 (http://www.exceluser.com/formulas/earlydates.htm). It also erroneously sees 1900 as a leap year, causing dates between 1 January 1900 and 28 February 1900 to be stored incorrectly (https://support.microsoft.com/en-us/help/214326/). Finally, if during import or processing, a certain column is formatted with the ‘Date’ data type—partial dates with a value only for year may become corrupted as Excel will interpret them as the number of days since 1 January 1900. For example, the year 1989 will become 11 June 1905. Users of Macintosh computers have similar but different issues with their versions of Excel (44).

Another common cause of date corruption has been the frequent use of work-arounds to indicate an imprecise date, such as using the first or midpoint of the year or month. Locally, these work-arounds are known by data managers but they frequently find their way to aggregated data such as on the Global Biodiversity Information Facility (GBIF), where they can be misunderstood (Figure 3). For dates where the year is not known (e.g. the label indicates ‘October’ or ‘summer’), various *ad hoc* solutions exist, such as a placeholder of ‘3000’ on the DoeDat transcription platform. These partial dates still have some utility, such as phenology, but they are not compatible with ISO 1806.

From the perspective of database integrity and computational efficiency, it would be ideal for dates to be stored as single dates in a single database column but this is incompatible with date ranges and partial dates.

Dates of many events can be documented with a specimen. Not only the collection date is the most obvious, but also the typification date, dates of expeditions, accession dates, determination dates and the date of transcription and digitization. Furthermore, there are other related dates, such as the birth and death dates of people, such as the collector and determiner, which can be used to validate these dates.

Some dates associated with specimens have no term in current standards. These include curatorial activities, such as received or communicated, as well as citation dates. By fully documenting the history of a specimen, the data becomes useful for more types of investigation, and through cross-validation of the data, the confidence in the data becomes stronger. The existence of dates and the length of date ranges are also useful metrics indicating the degree of transcription completeness.

Incidentally, the dwc:eventDate is one of the limited number of fields in Darwin Core that has a verbatim equivalent, dwc:verbatimEventDate. These verbatim fields are intended to be an exact transcription of what is written on the specimen to indicate to users how an interpreted standardized event date was derived (45). They can also be used to validate automatically generated data such as through OCR or to store data that could not immediately be converted to a standard such as ISO 1806, which may be two different use cases (Table 1).

#### Recommendations to standards organizations, developers and data scientists

All IT systems should support date ranges. Locally, they can be stored in a way that is easy to query, but for data exchange only ISO 1806 should be supported. Specimens without an explicit documented date of collection should be dated by other means, such as from itineraries of the collector. This step can be done automatically before the actual transcription takes place. However, interpretations of dates like this need to be documented. No digitized specimen record should lack some sort of date as, at the very least, the digitization date is available. Even a broad date range, such as knowing the century (e.g. in the shape of 1 January 1901/31 December 2000), is useful for validation and is sufficient for some use cases. Someone might be interested in all specimens in a certain collection from the 18th century, for instance. Many databases support generated columns, and this would not only be a way to maintain a single authoritative date field in a database but also provide efficient indexing for date-based searches and sorting. On the other hand, dates that lack a year are not compatible with ISO 1806 and should never be present in a standardized date field. Though such dates can be added to dwc:verbatimEventDate.

All dates associated with a specimen can be cross-validated and should be consistent. For example, a specimen cannot be determined or used as a nomenclatural type before it is collected. A specimen can only be collected within their lifetime. Furthermore, if dates are missing, then their possible range can be determined from all the other dates. If dates are inferred, this should be indicated in a human- and machine-readable manner to avoid creating a self-assuring cycle of validation.

A simple, machine-readable controlled vocabulary is required to indicate the origin of a date. Such a vocabulary might include the following elements: (i) verbatim transcription from the specimen label, (ii) interpretation from the specimen label, (iii) interpreted from date of expedition, (iv) date of a duplicate specimen, (v) interpreted from the sequence of collector numbers, (vi) interpreted from biographical details and (vii) interpreted from literature.

Dates are one of the most useful types of data associated with a specimen and also one of the easiest data elements to validate and cross-validate. Nevertheless, we have inherited a jumble of differently formatted dates in different implementations. This is one area where meticulous conformity to standards could make a significant contribution to interoperability.

### Where: geography

Geographic location is one of the three core elements of a natural history observation, and it is critical to associate a specimen with other information, such as climate, soil and land cover. The vast majority of specimens were collected before the advent of global positioning systems and the data written on these specimens are susceptible to large errors in comparison to what we are now used to. In many countries, particularly in the second half of the 20th century, biological recording has been based on national standard grid systems. These grid systems are based on a particular geographic projection and coordinate system and have a specific notation (e.g. Ordnance Survey National Grid, Belgian IFBL grid system). For example, the Ordnance Survey National Grid of Great Britain uses a grid laid on Airy 1830 ellipsoid that uses the Universal Transverse Mercator coordinate system with an origin to the south-west of Great Britain. The grid cells are then identifiable by letters denoting the 100 × 100-km grid cells and numbers for the cells within these. For example, NZ2085 identifies a 1 × 1-km grid cell with easting and northings of its south-western corner of 420 000 and 585 000 to the nearest meter.

Grid coordinates are generally converted to a grid centroid coordinate and error radius before they are shared on GBIF. This is misleading and degrades the value of this geographic information. Unfortunately, it has not been appreciated that all coordinate systems refer to an isosceles trapezoid on the Earth’s surface and not a true point. The conversion of grids to points is particularly regrettable because all points must be regridded to use them in species distribution models, but at that point the details of the survey have been lost as observations made in a grid will have been mixed with observations collected as points and radii. However, centroid-radius notation is so embedded in data collection that both systems must be accommodated.

Some geographic entities are clearly delimited and identifiable so can be specified with stable identifiers. These include not only political entities such as countries and counties but also entities of physical geography, such as mountains, islands, rivers and other landmarks. The boundaries of these entities vary in how fixed they are and political boundaries tend to change with time. However, identifiers for geographic entities could play a much greater role in disambiguating places than is currently the case.

Location information is useful in validation of other specimen information, constructing collector itineraries and for ecological research. Coordinates are in principle a good identifier but only if the coordinate system and datum are known. Coordinate precision is also critical, which if not given may be estimated based on the method of georeferencing used.

Georeferencing of specimens is a time-consuming form of data enrichment. It can take considerable research into other specimens, databases, maps and literature to estimate the collection location of a specimen and the margin of error that is given to it. Yet, if this research is not documented and the provenance is not recorded, it is possible that this work can be undone by someone not informed about the source of the georeferencing or an automated coordinate validation tool.

#### Recommendations to standards organizations and developers

Collection management systems and data standards should anticipate the use of local and national grid systems and have a means to validate them. Georeferencing tools should be available in the transcription system to ease and improve the transcription of geographic locations.

#### Recommendations to transcribers and data scientists

Local grid references should be documented in a verbatim coordinates field. National mapping agencies do change their coordinate systems, so clarity is also needed on which system is used. Users of Darwin Core should complete the dwc:verbatimCoordinateSystem and dwc:verbatimSRS fields.

Within institutional gazetteers stable identifiers should be linked to geographic entities. Sources of such identifiers are Geonames (https://www.geonames.org/), Getty Thesaurus of Geographic Names^®^ (https://www.getty.edu/research/tools/vocabularies/tgn/about.html), Marine Regions (http://www.marineregions.org/) and Wikidata (https://www.wikidata.org/). Use these identifiers to cross-reference locations and to validate coordinates. Share these data openly so that they can be used by others to validate their locations and build upon your work. Where georeferencing decisions are potentially controversial explain your reasoning in dwc:georeferenceRemarks.

### Which: collection number

Collection numbers or field numbers are identifiers for specimens or collecting events that are widely used in botany and to a lesser extent in zoology. They are indicated on the physical specimen and often refer to an entry in a field notebook with more detailed information concerning the collection event. They may refer to a single specimen’s collecting event or multiple specimens collected at the same time, in the same place and by the same people, for instance when duplicates are sent to multiple herbaria, also known as a gathering (42). In their simplest form, they may simply constitute numbers starting from 1 for the first specimen(s) collected. This means that there is no guarantee that these numbers are unique. Other information, such as who, where and/or when are required to uniquely identify a specimen and possibly its duplicates. Most collectors use their own format for their specimen numbers and there are no guarantees that these formats are utilized consistently. For instance, they may implement deviations of their standard approach as they encounter unusual situations.

Nevertheless, these numbers have more potential for analysis than simply a locally unique identifier (46). Often, specimen numbers are ordered by their time of collection so that the order of collecting can be inferred and an itinerary, approximated for the whole collecting trip. Missing data for certain specimens, such as when or where, can be inferred based on their position in the sequence of specimen numbers. However, this requires these numbers to be processed as numbers, whereas they often include additional non-numeric characters in various ways. These characters may not only be some sort of identifier for the collector or the collecting trip’s location but also part of the numbering protocol. Example collection numbers from the collection of Meise Botanic Garden include 25, SP07L26, 4674_BIS, 262A, 699^*^, 1874/12 and DDV/77/108.

Previously, the recommendation was made to separate non-numeric prefixes and suffixes from the actual (numeric) numbers (27). This occurs in other systems as well, such as BG-BASE™, a collection management system designed for use by botanic gardens. The standards of DwC and ABCD have only a single term for it: dwc:recordNumber and abcd:CollectorsFieldNumber. The problem with the split approach is that identifying the correct prefix and suffix is not always possible. It is also not clear within this approach how to deal with characters that are neither numbers nor letters. These may not only be used as delimiters (e.g. 1874/12) but also add another layer of uniqueness, e.g. 699^*^ for a distinct (but possibly similar) specimen to the one with 699 as a number. Some numbers will also have a combination of characters that cannot easily be separated into prefix, number and suffix (e.g. SP07L26).

#### Recommendations for transcribers and standards organizations

Collection numbers today are mostly transcribed in a verbatim manner and, if they are interpreted in some way, there is no standard as to how they are supposed to be interpreted. This is partially a consequence of how poorly they can be standardized. We would suggest differentiating between a verbatim and an interpreted data field for this property. The interpreted field should include no characters other than alphanumeric ones. This approach was taken in a previous report to improve finding matches between different transcriptions of the same specimen and should do the same for finding matches between identical numbers on different specimens and related numbers on similar specimens (27). This would facilitate matchmaking more than splitting up the data, as it reduces the interpretation made by the transcriber in how the split has to be made.

### Who: people

Many people can be associated with a specimen: the collector, curator, determiner, annotator, mounter, transcriber, digitizer, imager and georeferencer. For many reasons, these people are important to science. Knowing the person gives a degree of credibility to the specimen and its identity. The biographical data of the people can not only help validate data, but also credit the people for the work they have done (47). People often work as teams and this should be documented as well. Indeed, the order in which people’s names are written is important and needs to be maintained. The whole name of a person is relevant, including titles, prefixes and suffixes. These can be used to determine the gender, qualifications, relationships, organizational membership and profession and so are invaluable for disambiguation. For a specimen’s collector, ABCD has a ‘GatheringAgentsText’ field, where full name details could be written, but Darwin Core lacks a verbatim field for the collector or any other person associated with the specimen. In general, people are identified more completely and unambiguously on other academic and creative works, such as publications.

#### Recommendations for transcribers and standards organizations

Teams should always be broken down into individuals and their sequence preserved explicitly. Where available, the full verbatim name and the interpreted name should be recorded. Also, where available, people should be disambiguated with unique identifiers that link to their biographies.

#### Recommendations to software developers

Data entry systems should cross-validate data at the point of entry, such as cross-referencing collector biographies with collection date. Transcription systems should provide supplementary information to the transcribers to make informed decisions when trying to disambiguate people.

## Discussion

There are several reasons why it is important to improve the quality of transcribed label data and standardize it across institutions. Firstly, there is a large amount of time and money invested in transcription, whether through creation of citizen science crowdsourcing platforms or commercial transcription. Secondly, to fulfil the aims of digitization we need to provide the data that users need in a format suitable for their analysis. This inevitably means ensuring interoperability with other systems. Thirdly, once transcribed, these data are intended to last a long time, and they will hopefully be curated and improved. Lastly, perhaps the most dynamic post-transcriptional addition to a specimen record will be linking it to other information on taxa, people, other specimens, geography, etc. The ability to link data such as these is directly related to our ability to identify these entities clearly on labels.

A globally linked infrastructure of specimens is foreseeable in the near future (48). We already have the Global Biodiversity Information Facility where much of these data are available. However, the numerous papers on data cleaning and data quality attest to the need for further standardization and improvement. The responsibility for such improvements is shared by many institutions, professions and individuals, but in all cases there must be a collaborative effort to work with standards and improve them.

Finally, it is recommended that collecting practices are thoroughly reviewed so that digital data are generated on each specimen at, or soon after, the time of collection. Only then can the transcription of specimen labels be considered a legacy problem.
